# How much of the intraaortic balloon volume is displaced toward the coronary circulation?

**DOI:** 10.1016/j.jtcvs.2009.10.015

**Published:** 2010-07

**Authors:** Christina Kolyva, George M. Pantalos, John R. Pepper, Ashraf W. Khir

**Affiliations:** a Brunel Institute for Bioengineering, Brunel University, Middlesex, United Kingdom; b Cardiovascular Innovation Institute, University of Louisville, Louisville, Ky; c Royal Brompton Hospital, London, United Kingdom

**Keywords:** CBF, coronary blood flow, CPB, cardiopulmonary bypass, IAB, intraaortic balloon, IABP, intraaortic balloon pump, LV, left ventricular

## Abstract

**Objective:**

During intraaortic balloon inflation, blood volume is displaced toward the heart (V_tip_), traveling retrograde in the descending aorta, passing by the arch vessels, reaching the aortic root (V_root_), and eventually perfusing the coronary circulation (V_cor_). V_cor_ leads to coronary flow augmentation, one of the main benefits of the intraaortic balloon pump. The aim of this study was to assess V_root_ and V_cor_ in vivo and in vitro, respectively.

**Methods:**

During intraaortic balloon inflation, V_root_ was obtained by integrating over time the aortic root flow signals measured in 10 patients with intraaortic balloon assistance frequencies of 1:1 and 1:2. In a mock circulation system, flow measurements were recorded simultaneously upstream of the intraaortic balloon tip and at each of the arch and coronary branches of a silicone aorta during 1:1 and 1:2 intraaortic balloon support. Integration over time of the flow signals during inflation yielded V_cor_ and the distribution of V_tip_.

**Results:**

In patients, V_root_ was 6.4% ± 4.8% of the intraaortic balloon volume during 1:1 assistance and 10.0% ± 5.0% during 1:2 assistance. In vitro and with an artificial heart simulating the native heart, V_cor_ was smaller, 3.7% and 3.8%, respectively. The distribution of V_tip_ in vitro varied, with less volume displaced toward the arch and coronary branches and more volume stored in the compliant aortic wall when the artificial heart was not operating.

**Conclusion:**

The blood volume displaced toward the coronary circulation as the result of intraaortic balloon inflation is a small percentage of the nominal intraaortic balloon volume. Although small, this percentage is still a significant fraction of baseline coronary flow.

The intraaortic balloon pump (IABP) provides mechanical support to the heart in the preoperative, postoperative, and intraoperative surgical setting and in the medical setting in clinical conditions such as cardiogenic shock, myocardial infarction, and unstable angina.^1-4^ The IABP is also often used as a bridge to cardiac transplantation.^3^

The intraaortic balloon (IAB) starts to inflate immediately after left ventricular (LV) ejection and completes deflation before the onset of the following LV ejection. Accurate timing is essential for optimum IABP performance.

When the IAB is inflated it displaces blood volume upstream, toward the heart, increasing early diastolic aortic pressure and providing a boost to coronary blood supply. Similarly, when the IAB is deflated it draws blood volume downstream, away from the heart, reducing end-diastolic aortic pressure and thus LV afterload. Other beneficial effects related to these major outcomes are decreased LV volume and LV systolic work, reduced end-diastolic and peak-systolic aortic pressure, increased stroke volume, and improved cardiac output.^3^

This study focused on the phase of IAB inflation and more specifically on the blood volume displaced upstream during inflation. In vivo studies report increased peak diastolic and mean coronary flow velocity, and increased diastolic coronary flow velocity integral in healthy coronary vessels,^2,5-7^ but reports on the blood volume flowing through the coronary arteries as the result of inflation (V_cor_) are scarce. A microsphere study in sheep reports V_cor_ of 0.1 mL/min/g in myocardial tissue supplied by a nonstenotic artery,^8^ but there are no comparable data in humans.

The IAB is placed in the descending aorta, between the arch vessels and the renal arteries. During IAB inflation, blood volume is displaced upstream of the IAB tip (V_tip_), travels retrograde in the descending aorta, passes by the arch vessels, travels into the ascending aorta, arrives at the aortic root (V_root_), and eventually is directed into the coronary circulation as V_cor_. V_root_, V_cor_, and their relation to V_tip_ are not adequately reported in the literature. Accordingly, the aim of this study was to measure V_root_ in humans, as an indication of V_cor_. A more detailed in vitro study in a mock circulation was also conducted, in which the distribution of V_tip_ in the aortic arch, coronary branches, and compliant aortic wall was quantified.

## Materials and Methods

### In Vivo Data

The study population consisted of 10 patients (7 men; mean age 56 ± 5 years) who were supported by the IABP at least 2 days before open surgery for ventricular assist device placement. The protocol was approved by the Human Subjects Protection Program Office of the University of Louisville, and written informed consent was obtained from all patients. The data were available as part of an earlier study (American Heart Association award 0355187).

IAB catheters (Datascope Corp, Wayne, NJ) with a balloon size of 34 or 40 mL were inserted via the femoral artery and connected to the IABP (System 98 or CS100, Datascope Corp).

Patients were anesthetized according to a routine protocol, and anesthesia was maintained with isoflurane. Simultaneous aortic (P_ao_) and LV (P_LV_) pressure signals were recorded with a high-fidelity 5F dual-pressure-sensor catheter (Millar Instruments, Houston, Tex). Flow (Q_ao_) was measured at the same location as P_ao_ with a perivascular flow probe (Transonic Systems Inc, Ithaca, NY). P_ao_ and Q_ao_ were obtained at the aortic root, distal to the coronary arteries. Hemodynamic data and electrocardiograms were recorded at 200 or 400 Hz.

In all patients, data were recorded before ventricular assist device placement with IABP assistance frequencies of 1:1 and 1:2, and when the pump was on standby. Timing of inflation and deflation was based on the aortic pressure signal recorded internally by the pump with the fluid-filled catheter incorporated in the IAB.

For each pump setting, data were collected continuously for approximately 15 seconds, and a few minutes were allowed between successive measurements for hemodynamics to stabilize.

**Figure 1:**
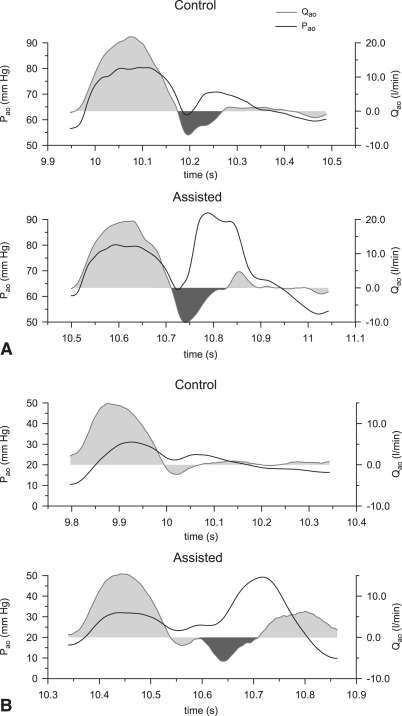
Hemodynamic waveforms in vivo during 1:2 IABP support for a control (*top*) and an assisted (*bottom*) beat in 2 different patients (A, B). P_ao_, aortic pressure (*solid black*); Q_ao_, aortic flow (*filled grey*). In cases of correctly timed inflation (A), V_root_ was derived by subtracting the intrinsic backflow of the control beat from the backflow of the assisted beat (both areas shaded in *dark grey*). In cases of late inflation (B), V_root_ was derived directly from the assisted beat by integrating the area shaded in *dark grey*.

For each patient, a single representative beat was selected for each condition. Figure 1 shows typical examples of P_ao_ and Q_ao_ recordings during a control and its following assisted beat from a 63-year-old male patient (Figure 1, *A*) and a 60-year-old male patient (Figure 1, *B*). As can be deduced from the onset of the rapid increase in P_ao_ during early diastole with respect to the incisura, Figure 1, *A,* corresponds to a case of well-timed inflation, whereas Figure 1, *B,* illustrates a case of fortuitous late inflation.

From the Q_ao_ measurements, V_root_ was calculated by integrating over time the negative peak during IAB inflation (Figure 1). V_root_ was normalized with respect to IAB volume to allow for comparing the results between patients assisted with different IAB sizes. Averages per beat were calculated for P_ao_, Q_ao_, and systolic P_LV_.

### In Vitro Data

A physiologic distribution of resistance and compliance was applied across a silicone aortic model (Ranier, Cambridge, UK) with 14 main branches (celiac, splenic and left and right coronary, carotid, subclavian, renal, femoral and deep femoral branches). Realistic values for terminal resistance and compliance of each branch were obtained from the model described by Stergiopulos and colleagues,^9^ after reducing their 55-branch model to 14 branches by summing resistances and compliances according to basic *in parallel* and *in series* electrical circuit concepts. Flow out of each branch was directed to a common drainage tube that was connected to an overhead water reservoir providing a head pressure of 10 mm Hg in the silicone model.

An extracorporeal LV assist device (BVS5000, Abiomed Inc, Danvers, Mass) was used to simulate the native heart in vitro. It was driven with water by a piston pump (Placepower, Norfolk, UK) and provided a cardiac output of 2.7 L/min at a heart rate of 60 beats/min, working close to its maximum capacity. The left ventricle of the artificial heart was connected to the aortic root of the aortic model, and the left atrium was connected to the overhead reservoir. The IABP was triggered by the piston pump.

Intraaortic balloons (Datascope Corp), sized 25 mL and 40 mL, were inserted via the left common femoral branch, advanced into the aorta until their tips were just distal to the subclavian branch, and connected to the IABP (System 97e, Datascope Corp).

Aortic pressure at the tip of the IAB (P_tip_) was recorded with a solid-state 7F sensor-tipped catheter (Gaeltec Ltd, Isle of Skye, UK). Flows (Q) at the tip of the IAB (Q_tip_) and through the carotid, subclavian, and coronary branches were measured with 20-, 10-, 8-, and 3-mm flowprobes, respectively (Transonic Systems Inc). Data were digitized and recorded at 500 Hz.

To assess the effect of the IAB inflation separately from the combined effect of IAB inflation and diastolic recoiling of the aorta, recordings were made with the IABP operating in 1:1 and 1:2 with and without the artificial heart. When the balloon was simply pumping against standstill conditions, the IABP was driven by a patient simulator (System 90 Series IABP Trainer, Datascope Corp) set at a heart rate of 60 beats/min. Steady-state intraaortic pressure was 66 mm Hg to simulate mean diastolic pressure in patients.

**Figure 2:**
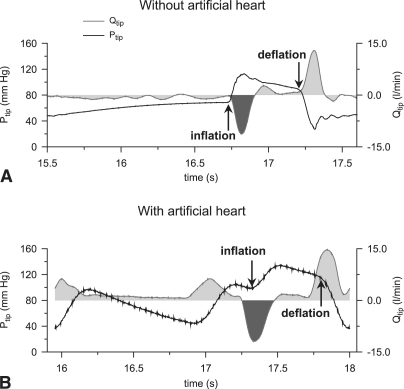
Pressure (P_tip_, solid black) and flow (Q_tip_, filled grey) in vitro during 1:2 IABP assistance. The onset of inflation and deflation (indicated by the *arrows*). A, No artificial heart is connected to the system. B, IABP is synchronized to the artificial heart. V_tip_ was derived by integrating the area shaded in *dark grey* in each case.

Multiple beats were analyzed for each condition. Figure 2 shows typical examples of P_tip_ and Q_tip_ recordings during 1:2 assistance without (top) and with the artificial heart (bottom) for a control and its following assisted beat.

The coronary, subclavian, and carotid Q measurements and Q_tip_ were integrated with respect to time as shown in Figure 2 (for Q_tip_) to derive the blood volume (V) displaced through the respective branches and upstream of the IAB tip due to inflation. By subtracting V_tip_ from the nominal balloon volume, we derived an approximation of the volume displaced downstream. The volume displaced upstream of the IAB tip, V_tip_, without going through the coronary or arch branches, is stored in the compliant aortic wall and is referred to as “V_compliance_.”

### Statistical Analysis

Data are expressed as mean ± standard deviation. For the in vivo data, results between assistance frequencies 1:1 and 1:2 and pump off were compared with analysis of variance with repeated measures, followed by contrast analysis (SPSS v 15.0; SPSS Inc, Chicago, Ill). Because of the small patient population, a more elaborate statistical analysis taking into account possible interactions from factors such as balloon size, could not be performed.

For the in vitro data, statistical comparisons were only made with and without the artificial heart, for each balloon size and assistance frequency, with unpaired *t* tests. Comparisons between the assistance frequencies or the balloon sizes were not considered to be of important clinical information and were not performed.

## Results

### In Vivo Results

#### Hemodynamic signals

Diastolic aortic pressure augmentation during inflation was demonstrated by a steep increase in the P_ao_ signal of the assisted beat (Figure 1) and coincided with a negative peak in Q_ao_, induced by blood volume displacement toward the aortic root. This peak was either overlapping with aortic retrograde flow (Figure 1, *A*) or followed immediately after as a distinct second negative peak (Figure 1, *B*).

#### Volume displacement

During 1:1 assistance, V_root_ was 6.4% ± 4.8% of the IAB volume and increased to 10.0% ± 5.0% during 1:2 assistance, but the difference between the 2 assistance frequencies was not statistically significant (*P* = .16).

#### Hemodynamic parameters

With 1:1 assistance, mean P_ao_ increased by 11.2% (61.6 ± 16.5 mm Hg vs 68.5 ± 16.4 mm Hg, *P < .*005). The increase was 20.2% with 1:2 assistance (58.8 ± 17.1 mm Hg vs 70.7 ± 17.9 mm Hg, *P < .*0001) and was significantly higher with respect to 1:1 assistance (*P < .*05). Mean systolic P_LV_ significantly decreased during 1:1 assistance by 8.7% (76.6 ± 21.0 mm Hg vs 69.9 ± 19.8 mm Hg, *P < .*0005), and similarly during 1:2 assistance there was a decrease of 4.6% (73.8 ± 22.6 mm Hg vs 70.4 ± 20.7 mm Hg, *P < .*05). Mean Q_ao_ increased significantly during 1:1 assistance by 26.4% (2.92 ± 1.06 L/min vs 3.69 ± 1.29 L/min, *P < .*01) and by 13.0% (2.99 ± 1.27 L/min vs 3.38 ± 1.25 L/min, *P < .*05) during 1:2 assistance.

### In Vitro Results

#### Hemodynamic signals

Early diastolic pressure augmentation was evident in the P_tip_ recording (Figure 2, *B*) during IAB inflation, similar to Figure 1. The same finding can also be observed in Figure 2, *A,* but with no heart function. Both with and without the artificial heart, inflation was accompanied by a peak in Q_tip_ that was caused by fluid volume displacement toward the aortic root.

#### Volume displacement

**Figure 3:**
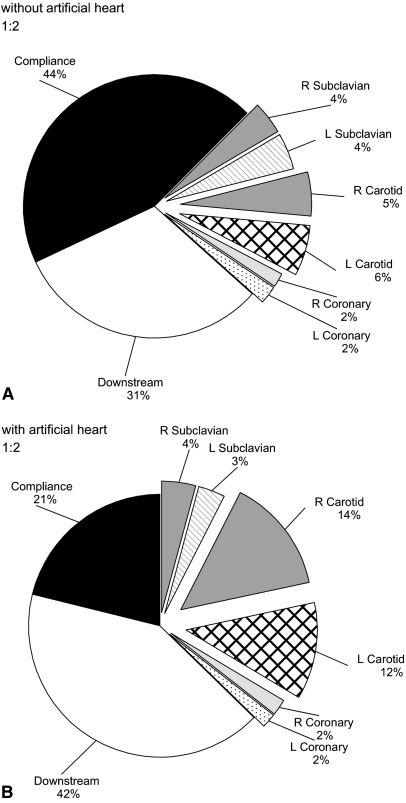
Percent distribution of the total volume displaced upstream and downstream of a 25-mL IAB during inflation at 1:2 assistance without (A) and with (B) the artificial heart connected to the in vitro setup. *L,* Left; *R,* right.

Figure 3 shows the percent distribution of IAB-induced flow during inflation in the coronary, carotid, and subclavian arteries, compliant aortic wall, and downstream of the balloon without and with heart function simulated by the artificial heart. The results correspond to a 25-mL IAB at an assistance frequency of 1:2. Overall, when the balloon was counterpulsating with the artificial heart, less fluid volume was stored in the compliant aortic wall (5.3 vs 11.1 mL) and more was displaced downstream of the balloon (10.4 vs 7.9 mL) and overall through the arch and coronary branches (9.3 vs 6.0 mL). The volume displaced through both left and right coronary branches was 1.0 mL without the artificial heart and 0.9 mL with the artificial heart.

**Figure 4:**
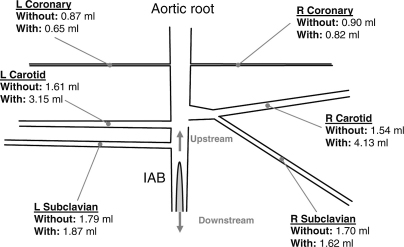
Upper portion of the artificial aorta showing the tip of the IAB and the approximate locations where flow was measured in the coronary and arch branches. The fluid volume that passed through each branch in vitro because of the inflation of the 40-mL IAB at 1:1 frequency is noted both for the cases with and without artificial heart connected to the system. *L,* Left; *R,* right; *IAB,* intraaortic balloon.

Figure 4 shows the volume distribution in milliliters for a 40-mL balloon during 1:1 IABP support without and with heart function simulation. Similar to the findings illustrated in Figure 3, with the artificial heart, less fluid volume was stored in the compliant aortic wall (14.0 vs 21.8 mL) and more fluid volume was displaced downstream of the balloon (13.8 vs 9.8 mL) and through the arch and coronary branches (12.2 vs 8.4 mL). The total volume displaced through the coronary branches was 1.8 mL as opposed to 1.5 mL without the artificial heart. Qualitatively, these findings remained consistent between different balloon sizes and different assistance frequencies (Table 1).

## Discussion

This study shows that the blood volume displaced in vivo toward the coronary circulation during IAB inflation is no more than 10% of the nominal balloon volume. These results are in agreement with our in vitro data, demonstrating that the fluid volume passing through the coronary branches during inflation is less than 5% of the IAB volume. The in vitro investigation further revealed that a large percentage of the fluid volume displaced upstream of the IAB tip during inflation is stored in the compliant aortic wall. This percentage was approximately 2 times higher when the artificial heart was not simulating the native heart function.

### Augmentation of Coronary Perfusion During Inflation

Both the in vivo and in vitro results show that V_root_ and V_cor_, respectively, are only a small fraction of the total balloon volume, with the rest of V_tip_ being distributed between the arch branches and stored in the compliant aortic wall. In humans, V_root_ was 6.4% during 1:1 IABP support and 10.0% with 1:2 IABP support. In vitro and with the artificial heart simulating heart function, V_cor_ was even smaller, 3.7% and 3.8%, respectively.

Although V_root_ in vivo and V_cor_ in vitro are small, an additional blood volume of 1 to 2 mL per beat in the coronary circulation is a significant increase to baseline coronary blood flow (CBF). In humans with healthy coronary vessels, a mean CBF of 200 mL/min and heart rate of 75 beats/min^10^ provide approximately 2.5 mL of blood to the coronary circulation during each heart cycle. Therefore an augmentation of 1 to 2 mL resulting from the IABP represents a significant increase in CBF.

Augmentation of coronary perfusion during IABP support has been quantified in a microsphere study in sheep.^8^ The authors measured myocardial perfusion using microspheres in myocardial regions supplied by an obstructed artery or by normal vessels. Measurements were taken at control conditions and during IABP counterpulsation with a 40-mL IAB. In the regions supplied by healthy coronary vessels, CBF was 0.55 mL/min/g at control and 0.65 mL/min/g during counterpulsation. For an average heart weight of 300 g, coronary perfusion would therefore be 2.75 mL at baseline and 3.25 mL with IABP support. Despite the differences in the experimental settings that make a direct quantitative comparison of these results with ours difficult, both studies are in agreement that the percentage of IAB volume that reaches the coronary circulation is small and that this small percentage is a large contribution to baseline coronary perfusion.

Our in vitro measurements were obtained in unobstructed branches; therefore, it would be difficult to extrapolate the above observations to stenotic coronary vessels. In the presence of a stenosis, the effect of the IABP on CBF depends inversely on the severity of the stenosis, with the potential of even a reduction in CBF in cases of severe stenoses.^11-14^ Our findings on V_root_ in vivo and V_cor_ in vitro projected into the surgical setting suggest that IABP assistance may be particularly beneficial after coronary artery bypass grafting to improve coronary perfusion in the immediate postoperative period, when the heart is stunned after cardioplegia, cardiopulmonary bypass (CPB), and the consequent inflammatory cascade.

### Intraaortic Balloon Volume Distribution

V_tip_ has been quantified in vitro in a straight latex tube for different IAB sizes, and it was approximately 57% of the nominal balloon volume.^15^ Head pressure was 24 mm Hg, and the IAB was operated from standstill. In view of the different experimental settings, these results are not substantially different from those of the present study, in which V_tip_ was 72.6% at 1:1 and 68.1% at 1:2 without the artificial heart and 63.1% at 1:1 and 62.0% at 1:2 with the artificial heart.

Figures 3 and 4 and Table 1 show the detailed distribution of V_tip_ at different aortic branches in vitro. Corresponding qualitative data are not available in vivo, but from published studies on the differences between nonpulsatile and IABP-induced pulsatile perfusion during CPB, it is possible to qualitatively discern the net effect of the IABP on the perfusion of different organs and vascular beds. For example, increased tissue oxygen pressure in the renal medulla and decreased local lactate levels have been found in CPB with IABP.^16^ On the other hand, CPB without IABP has been associated with renal hypoxia and acidosis.^17,18^ Likewise, progressive systemic arterial vasoconstriction has been demonstrated in the absence of IABP, leading to reduced perfusion and acidosis.^19^ Similar findings have emerged from studies of the splanchnic circulation, with reduced frequency of elevated amylase levels observed in patients receiving CPB with IABP.^20^ IABP during CPB preserves the liver, decreasing aspartate aminotransferase leakage.^21^ More recently, the benefit of IABP during CPB has been investigated in an elderly population by Onorati and colleagues.^22^ A significant improvement in respiratory function was found, and it was concluded that IABP-induced pulsatile flow significantly improves whole-body perfusion.

### Volume Storage in the Aorta In Vitro

It was not possible to determine the distribution of stored volume along the compliant aortic wall in vitro, but, merely because of size, it can be speculated that the larger storage capacity lies in the upper aorta, in the portion upstream of the IAB tip. The length of this segment in the silicone aorta was 17 cm, with an average diameter of 24 mm. Measurements with ultrasonic crystals of the diameter of the upper descending sheep aorta during IAB counterpulsation showed an increase of 4.4% between systolic diameter and maximum diastolic diameter during inflation.^23^ An increase of 4.4% in the mean diameter of the 17-cm segment of the silicone aorta can be translated to an increase of 6.5 mL of its total volume capacity during inflation. This volume is not dissimilar to the results of our in vitro study, showing volumes of 5.79 mL during 1:1 assistance and 5.29 mL during 1:2 assistance stored in the compliant aortic wall during the inflation of a 25-mL IAB. The corresponding volumes for a 40-mL IAB are larger, most likely because the aortic model was overstretched during IAB inflation in a way that would have been prevented in vivo by using a smaller IAB size.

### Effect of Heart Function on Intraaortic Balloon Counterpulsation

When the IAB was counterpulsating with the artificial heart, V_tip_ was smaller than when operating without the artificial heart. However, overall more volume was distributed to the arch and coronary branches and less was stored in the compliant aortic wall. This effect is clearly demonstrated in Figures 3 and 4 and in Table 1.

Because there is minimal diastolic or no flow in the aorta during IAB inflation in both in vitro setups, this difference in volume distribution can be associated with a difference in compliance.^3^ When IAB inflation follows a cardiac systole, because of the Windkessel effect taking place in the aorta during diastole, the elastic aortic wall will be recoiling at the same time the inflating balloon is trying to expand it. As a result of these opposing actions, aortic compliance is lower and the fluid volume that can be stored in the aortic wall is less than the volume that can be stored when the aorta can freely expand during inflation. The fluid volume not stored in the compliant aortic wall in the presence of heart function is then distributed elsewhere (Figures 3 and 4). The discharging of the fluid volume stored in the compliant aortic wall is demonstrated in Figure 2, *A,* by the exponential increase in P_tip_ between 15.5 and 16.5 seconds.

This result indirectly underlies the importance of arterial compliance on IABP performance. These findings are in agreement with previous studies stating that the efficiency of the IAB is limited in highly compliant aortas.^24^

The in vitro experiment without the artificial heart simulated the clinical setting of CPB and aortic crossclamping, with the IABP providing pulsatile flow. Although standard CPB with nonpulsatile flow is routinely used in surgical practice, the added benefits of pulsatile perfusion include improvement in organ perfusion caused by a reduction of vasoconstrictive reflexes, improved oxygen consumption, and a reduction of acidosis.^19^

### Effect of Assistance Frequency In Vivo

The blood volume displaced toward the coronary circulation during the assisted beat of the 1:2 support is less than the combined volume displaced by 2 consecutive beats during 1:1 support. Although the results are not statistically significantly different for this patient population, it is possible that in a larger population differences would be more pronounced.

### Methodological Considerations and Limitations

It is expected that the reduction in systolic P_LV_ during 1:1 assistance in patients will induce coronary vasoconstriction because of the reduced myocardial oxygen demand,^25^ and therefore reduce CBF, whereas on the other hand volume displacement caused by inflation simultaneously tends to increase it. This mechanism could not be duplicated in vitro, and it is difficult to predict how this practically affected V_cor_. Other in vivo pathologic indications for using the IABP, such as cardiogenic shock and unstable refractory angina, were also not simulated, because they involve sympathetic and parasympathetic nervous activities that could not be replicated in vitro.

## Conclusions

The blood volume displaced toward the coronary circulation as the result of IAB inflation is only a small percentage of the nominal IAB volume. However small this percentage might be compared with the IAB volume, it is still a significant percentage of baseline coronary perfusion.

## Figures and Tables

**Table 1:** Mean volume distribution for the 25-mL and 40-mL balloons

	Balloon 25mL				Balloon 40mL			
	1:1		1:2A		1:1		1:12A	
Volume (mL)	No heart	Heart	No heart	Heart	No heart	Heart	No heart	Heart
R subclavian	1.09 ± 0.10∗	0.98 ± 0.08	1.02 ± 0.15	1.05 ± 0.17	1.70 ± 0.15	1.62 ± 0.32	1.49 ± 0.16∗	2.03 ± 0.31
L subclavian	1.21 ± 0.12∗	0.95 ± 0.19	1.12 ± 0.11∗	0.83 ± 0.13	1.79 ± 0.14	1.87 ± 0.39	1.65 ± 0.21	1.57 ± 0.27
R carotid	0.96 ± 0.03∗	3.45 ± 0.06	1.35 ± 0.03∗	3.54 ± 0.08	1.54 ± 0.05∗	4.13 ± 0.04	1.94 ± 0.12∗	4.59 ± 0.06
L carotid	1.00 ± 0.03∗	3.04 ± 0.14	1.55 ± 0.03∗	2.98 ± 0.03	1.61 ± 0.05∗	3.15 ± 0.08	2.11 ± 0.04∗	3.37 ± 0.05
R coronary	0.47 ± 0.01	0.48 ± 0.01	0.43 ± 0.01∗	0.50 ± 0.01	0.90 ± 0.01∗	0.82 ± 0.01	0.75 ± 0.01∗	0.85 ± 0.01
L coronary	0.58 ± 0.00∗	0.44 ± 0.01	0.56 ± 0.01∗	0.44 ± 0.01	0.87 ± 0.02∗	0.65 ± 0.01	0.74 ± 0.05∗	0.66 ± 0.01
Upstream	17.39 ± 0.92∗	15.13 ± 0.89	17.14 ± 1.10∗	14.61 ± 0.20	30.22 ± 2.18∗	26.25 ± 0.90	27.10 ± 1.45	26.18 ± 0.89

Volume (in milliliters) displaced into the arch and coronary branches and upstream of the IAB tip in vitro as the result of IAB inflation at different assistance frequencies, without (No heart) and with (Heart) the artificial heart. Mean values for the subclavian branches and aorta are based on 16 measurements, whereas 8 measurements are available for the other branches.

∗ *P < .*005, comparing no heart with heart. *R,* Right; *L,* left.
